# Kinetic Analysis of Pyrolysis and Thermo-Oxidative Decomposition of Tennis String Nylon Wastes

**DOI:** 10.3390/ma14247564

**Published:** 2021-12-09

**Authors:** Haibo Wan, Zhen Huang

**Affiliations:** 1 Department of Physical Education, Tianjin University of Commerce, Tianjin 300134, China; wanhaibo@tjcu.edu.cn; 2 Department of Packaging Engineering, Tianjin University of Commerce, Tianjin 300134, China

**Keywords:** nylon-6 tennis string wastes, thermal degradation, thermogravimetric analysis, isoconversional kinetics

## Abstract

Thermal degradation of nylon-6 tennis string nylon wastes in inert nitrogen and air atmospheres was investigated by means of multiple heating-rate thermogravimetric analyses. The results obtained under the heating rates of 5–20 K/min are compared in terms of degradation feature and specific temperature for two atmospheres. Using nonisothermal data, kinetic analysis was thoroughly conducted using various isoconversional model-free methods, including Starink, Madhusudanan–Krishnan–Ninan, Tang, Coats–Redfern, and Flynn–Wall–Ozawa methods. With these kinetic analysis methods, the activation energy over the entire degradation process was successfully calculated. By means of the model-fitting master-plots method, the first-order chemical reaction model was determined to be the most appropriate mechanism function for describing pyrolysis and oxidative thermal degradation of nylon-6 waste. Using kinetic parameters, satisfactory matching against experimental data resulted using the Coats–Redfern method for both cases. Furthermore, thermodynamic parameters such as changes in entropy, enthalpy, and Gibbs free energy during thermal degradation processes were evaluated.

## 1. Introduction

Today, tennis is a very popular sport all around the world and tennis balls, rackets, and strings along with other auxiliary supplies are drastically consumed. However, tennis produces various wastes such as tennis balls, rackets, and strings in considerable amounts as they are readily worn out under high energy and high-speed shots during tennis. Among various tennis string materials, nylon-6 is the most commonly used polymer in order to achieve the best performance in tennis playing. Nylon-6 is one kind of polyamide characterized by the amide group (–NHCO–) and it is one of the most widely used engineering plastic in many industrial fields including building, home textile, automotive and electrical industries since it possesses good chemical stability, high mechanical strength, and elasticity. Furthermore, it is extensively used to fabricate tennis string because of its excellent wear resistance meeting the requirements of tennis sport to a great extent.

In the present work, nylon-6 tennis string wastes in Tianjin, China, were collected for inert and oxidative thermal degradation considerations. It is well-known that pyrolysis and combustion are two effective methods to chemically converse plastic wastes into valuable substances for sustainable energy development [1,2]. To date, a number of attempts have been made to study pyrolysis and oxidative thermal degradations of nylon-6 and its wastes [3,4,5,6,7,8,9,10,11,12]. Principally, nylon-6 has been reported to thermally degrade into ε-caprolactam monomer through a chain end backbiting process or a ring transition state formed within polymer chains [3,4,5] and a yield as high as 83–85 wt.% of ε-caprolactam could be obtained [6,7]. Such recovery of high ε-caprolactam yield may become worse when dealing with nylon-6 wastes [8,9]. Chaihad et al. [8] studied the catalytic effect of calcined scallop shell (CSS) on pyrolysis of nylon-6 fishing net waste and found that the decomposition temperature can be decreased by 40–60 K in the presence of catalysts but the yield of ε-caprolactam has reduced to be 66 wt.%. Likewise, Kim et al. [9] performed a pyrolysis study of teabag waste at 400 and 700 °C and reported that the highest caprolactam yield has turned to be 59.2 wt.%. In contrast, the pyrolysis product composition may become diverse when changing pyrolysis conditions [10,11]. Bozi and Blazsó [10] studied the effect of acidic Y zeolite catalyst on nylon-6 pyrolysis and reported that the pyrolysis product may become mainly dihydro-azepine isomers instead of ɛ-caprolactam via *cis*-elimination or intramolecular rearrangement of amide groups and more aromatics and light unsaturated hydrocarbons will be yielded when using greater amounts of zeolite. Interestingly, Pannase et al. [11] reported that the condensed pyrolytic oil of fresh nylon-6 ropes, obtained at 823 K under 5 K/min and 873 K under 10 K/min and analyzed via Fourier transform infrared spectroscopy (FTIR), shows a high resemblance with conventional fuel such as diesel.

Apart from the study of degradation product compositions, kinetic analysis of thermal degradation of nylon-6 has also been attempted in terms of kinetic triplet parameters of as apparent activation energy *E_a_*, pre-exponential factor *A* and reaction mechanism model function *f*(α) using isothermal thermogravimetric (TG) data or dynamic non-isothermal TG data [4,5,7,11,12]. Holland and Hay [4] studied the pyrolysis of nylon-6 and reported an *E*_a_ calculated at 190 kJ/mol along with the first-order reaction for pyrolysis of fresh nylon. Similarly, Bockhorn et al. [12] thoroughly investigated the thermal degradation kinetics of nylon-6 in helium with/without catalyst under dynamic and isothermal conditions. Their works showed an *E*_a_ of 211 kJ/mol, log*A* of 15.0 min^−1^ and a reaction order of 0.81 for nylon-6 over the dynamic degradation process while in the case of isothermal condition nylon-6 had a lower *E*_a_ of 205 kJ/mol, lower log*A* of 14.5 min^−1^, and higher reaction order of around 1.0. As a comparison, the *E*_a_ was greatly reduced to 113 kJ/mol using a basic catalyst of NaOH/KOH eutectic mixture, along with log *A* = 8.7 min^−1^ and a reaction order of *n* = 1.06. However, with an acid catalyst of H_3_PO_4_, the *E*_a_ was also reduced to *E*_a_ = 164 kJ/mol, together with log *A* = 14.0 min^−1^ and a reaction order of *n* = 1.9. A rather similar *E*_a_ value of 205 kJ/mol has also been reported in the work of Pannase et al. [11], where nylon-6 pyrolysis was performed via model-free Coats–on redmine Redfern (CR), Flynn–Wall–Ozawa (FWO), and Starink (SK). However, quite distinctly, they reported that the degradation reaction is controlled by a contracting sphere R3 model other than the chemical order model identified by using the master-plot method [11]. 

Furthermore, kinetic analysis studies for pyrolysis of nylon-6 wastes have also been conducted and can be referred to in two works [5,7]. Kim et al. [5] studied pyrolysis of nylon-6 fresh and waste fishing nets and differentially obtained an average *E_a_* of 266 and 220 kJ/mol for fresh fishing nets and waste fishing nets, respectively. They also reported the reaction order of 0 or 1 for fresh nylon and waste nylon, respectively. Likewise, Eimontas et al. [7] reported that through isoconversional model-free kinetics analysis, the overall averaged activation energies were estimated at 112 kJ/mol for waste fishing nets and 158, 230, 197, 201, and 220 kJ/mol for waste fishing nets added with 2.5, 5, 10, 20, and 50 wt% ZSM-5 zeolite, respectively. At the same time, they also attempted the distributed activation energy model (DAEM) to plot thermogravimetry and differential thermogravimetry (TG/DTG) curves, leading to a high prediction to experimentally obtain multiple heating-rate TG and DTG curves [7]. However, more studies are needed to carry out about pyrolysis of nylon-6 wastes considering the extensive consumption of nylon-6 in various industrial fields. Once thermal degradation characteristics and kinetic parameters are available, not only would thermal degradation processes be predicted over the entire temperature range, but also thermal degradation reactors might be designed for processing massively accumulated nylon-6 wastes. 

The objectives of the present work are: (1) thermal characteristics and degradation kinetics of nylon-6 string wastes under inert nitrogen and oxidative air atmospheres are compared by means of thermogravimetric analysis. (2) The non-isothermal mass loss data obtained are kinetically analyzed to determine the kinetic parameters of *E*_a_, ln*A*, and *f*(α) with a number of isoconversional kinetic analysis methods and the calculation results with these methods are discussed in detail. The variation in activation energy with conversion can be used to reveal the complex nature of the thermal degradation reaction. (3) The model-fitting master-plots method has been attempted to scan the most appropriate mechanism model for describing oxidative or inert thermal degradation processes of nylon-6 tennis string wastes and the first-order chemical reaction model is found to be obeyed during thermal degradation processes. With these kinetic parameters, rebuilding the mass conversion curves has been performed, consequently resulting in satisfactory predictions against experimental results. (4) Finally, thermodynamic functions such as changes in entropy, enthalpy, and Gibbs free energy during pyrolysis degradation have also been quantified by assuming the transition state theory. The current work provides detailed information about thermal degradation behaviors and kinetic triplet parameters necessary for designing a chemical reactor to thermally dispose of nylon-6 wastes.

## 2. Materials and Analysis Methods 

### 2.1. Materials

Tennis string waste, from the same brand (^®^Taan of Shenzhen Taiang Industrial CO., Ltd., Shenzhen, China) was collected locally in Tianjin, China. The collected filaments were washed using distilled water and sun-dried in open air. Then, the dried sample after cutting was milled with a grinder and meshed to less than 100 um powder. The resultant powder was dried at 400 K in a controlled oven for 20 h to remove moisture residual and finally kept in an airtight container for subsequent measurements. 

### 2.2. TG Analysis

The powdery waste sample of 3–4 mg for each run was subjected to a thermogravimetric analyzer (DTG-60, Shimadzu, Japan) for thermal degradation measurements and differential thermal analysis (DTA) under a constant heating rate mode. The purging gas used was air or N_2_ at a flow rate of 30 cm^3^/min. The temperature range programmed was spanned. Non-isothermal TGA data were yielded from ambient up to 850 K under 5, 10, 15, and 20 K/min and the DTG data were automatically obtained with the analyzer software.

Fourier transform infrared (FTIR) analysis was performed on nylon-6 tennis filament waste samples via a Bruker Alpha-H infrared spectrophotometer and FTIR data acquired varied in the wavelength range of 400–4000 cm^−1^. FTIR results obtained with 32 scans for each run can be used to identify the chemical structure of real-world nylon-6 waste samples. The powder waste sample after being further milled with a mortar and pestle was mixed with KBr at a mass ratio of 5:95 and then pressed into a transparent sample disc for subsequent FTIR measurements.

### 2.3. Kinetic Analysis

Deep analysis of thermal degradation kinetics is a very useful tool for securing the thermal degradation process and explore degradation reaction mechanisms. Theoretical kinetics analysis has been commonly performed using isoconversional model-free and model fitting methods and the thermal degradation of various solids can be accurately characterized by using kinetic triplets of the activation energy (*E*_a_), the pre-exponential factor (*A*), and the reaction mechanism function (*f*(*α*)) [13,14]. With the isoconversional method, the *E*_a_ values are calculated as a function of the degree of mass conversion (*α*). Using the model-fitting method, the most appropriate reaction mechanism function (*f*(*α*)) can be properly found based on the *E*_a_ values, and the third kinetic parameter A can be readily determined thereafter. With these kinetic parameters, thermal degradation TG and DTG curves can be predicted and necessary information for subsequent industrial applications can be provided. Therefore, model-free and model-fitting methods are both attempted for analyzing thermal degradation of tennis string waste.

Generally, thermal reaction process of plastic solids may be described by the progress of degradation (*dα*/*dt*) as a function of time and temperature given below:

(1) dα/dt=k(T)⋅f(α)

where *k*(*T*) is the rate constant of thermal degradation reaction and is usually written as the Arrhenius equation *k*(*T*) = *A*·exp(-*E*_a_/*RT*), in which *E_a_*(J/mol) is the activation energy to satisfy degradation reaction. *A* (min^−1^) is the pre-exponential factor, *T* (K) is the absolute temperature and *R* is the universal gas constant (8.314 J/mol K). Furthermore, *f*(*α*) stands for a reaction mechanism function for describing the kinetic thermal degradation, and *α* is set to be the extent of mass conversion defined as:

(2) $$\alpha =\frac{m_{0}-m_{t}}{m_{0}-m_{\infty }}$$

where *m_0_*, *m_t_*_,_ and *m*_∞_ are the initial sample mass, the sample mass at degradation time *t*, and the final sample mass, respectively. So far, the isoconversional analysis method has been the most commonly used for thermal degradation kinetics and then non-isothermal temperature program is conducted at a constant heating rate mode with *β* defined as *β* = *dT*/*dt*. Subsequently, Equation (1) can be transformed as:

(3) $$\frac{d\alpha }{dT}=\left(\frac{A}{\beta }\right)\exp\left(-\frac{E_{a}}{RT}\right)\cdot f(\alpha )$$

Readily, an integral form of reaction mechanism function, *g*(*α*), can be deduced from Equation (3) by rearrangement and integration, given as:

(4) $$g(\alpha )=\int_{0}^{\alpha }\frac{d\alpha }{f(\alpha )}=\frac{A}{\beta }\int_{0}^{T}\exp(-E_{a}/RT)dTT$$

As for describing the thermal degradation mechanism, a number of reaction model functions in differential form of *f*(*α*) and integral form of *g*(*α*) are available [14] and some of them are presented in Table 1 in the present work.

#### 2.3.1. Model-free method

In Equation (4), a well-known temperature integral function is involved and can be further transformed by setting *x* = *E_a_*/*RT* and $p(x)=\int_{x}^{\infty }\frac{e^{-x}}{x^{2}}dx$ as:

(5) $$g(\alpha )=\int_{0}^{\alpha }\frac{d\alpha }{f(\alpha )}=\frac{A}{\beta }\int_{0}^{T}\exp\left(-\frac{E_{a}}{RT}\right)dT=\frac{AE_{a}}{\beta R}\int_{x}^{\infty }\frac{e^{-x}}{x^{2}}dx=\frac{AE_{a}}{\beta R}p(x)$$

However, there is no analytical answer to solve *p*(*x*), and thus numerous approximate solutions or numerical regressions have been proposed such as Starink (SK) [15], Tang et al. (Tang) [16], Madhusudanan–Krishnan–Ninan (MKN) [17], Coats–Redfern (CR) [18], and Flynn–Wall–Ozawa (FWO) [19,20]. They are detailed as given below:

(6) $$\mathrm{SK}\;\mathrm{method}:\;\ln\left(\frac{\beta }{T^{1.92}}\right)=\ln\left[\frac{AR}{g(\alpha )}{\left(\frac{E_{a}}{R}\right)}^{-0.92}\right]-1.0008\frac{E_{a}}{RT}-0.312$$

(7) $$\mathrm{Tan}g\;\mathrm{method}:\;\ln\left(\frac{\beta }{T^{1.894661}}\right)=\ln\left[\frac{A}{g(\alpha )}{\left(\frac{E_{a}}{R}\right)}^{-0.894661}\right]-1.00145033\frac{E_{a}}{RT}-0.37773896$$

(8) $$\mathrm{MKN}\;\mathrm{method}:\;\ln\left(\frac{\beta }{T^{1.884318}}\right)=\ln\left[\frac{A}{g(\alpha )}{\left(\frac{E_{a}}{R}\right)}^{-0.884318}\right]-1.001928\frac{E_{a}}{RT}-0.389677$$

(9) $$\mathrm{CR}\;\mathrm{method}:\;\ln\left(\frac{\beta }{T^{2}}\right)=\ln\left[\frac{AR}{E_{a}g(\alpha )}\right]-\frac{E_{a}}{RT}$$

(10) $$\mathrm{FWO}\;\mathrm{method}:\;\log\beta =\log[\frac{AE_{a}}{Rg(\alpha )}]-2.315-0.4567\frac{E_{a}}{RT}$$

Currently, these integral methods have been extensively used to analyze non-isothermal TG data and then isoconversionally calculate activation energy at any arbitrary mass conversion level [11,21,22,23,24,25]. Using these methods, the left term is conveniently plotted against 1/T and a straight line will result for each conversion level, from whose slope the activation energy *E*_a_ can be easily evaluated over the whole conversion range. As such, these methods are thought of as model-free methods since they do not consider any specific chemical reaction model. 

#### 2.3.2. Model-Fitting Method

As we can see, the above-given model-free methods can rather conveniently result in mass conversion dependent *E*_a_ but they are not directly related to the identification of the thermal degradation model. On the contrary, the model-fitting method has been frequently applied for such an aim. Among various model-fitting methods, one of the most frequently reported is the master-plots method given by Gotor et al. [26] to approximate the reaction model to the maximum extent. To apply this method, a reference has been introduced by defining an integral function *g*(*α*) at *α* = 0.5:

(11) $$g(0.5)=\frac{AE_{a}}{\beta R}p(x_{0.5})$$

where *x_0_._5_* equals *E_a_*/*RT_0_._5_* and *T*_0_._5_ is a specific temperature set at *α* = 0.5. Based on Equations (5) and (11), a mathematical expression to represent the most common master-plots method [11,21,22,26], named the G-master-plots method for simplicity, can be deduced as follows:

(12) $$\frac{g(\alpha )}{g(0.5)}=\frac{p(x)}{p(x_{0.5})}$$

where the right and left terms are experimental and theoretical plots, respectively. Clearly, all *g*(*α*)/*g*(0.5) and *p*(*x*)/*p*(*x*_0_._5_) curves are condensed to 1 at *α* = 0.5. By respectively drawing experimental *p*(*x*)/*p*(*x*_0_._5_) values versus *α* and theoretical *g*(*α*)/g(0.5) values versus *α* of various reaction models listed in Table 1, the matching degree of the considered reaction model against experimental data can be evaluated, and the reaction model with the best fitting among all the models in Table 1 can be determined as the kinetic reaction model of thermal degradation of nylon-6 tennis string waste. To make Equation (12) work, a fourth-order expression given by Senum and Yang [27] has been applied to evaluate the reaction model and such a high-precision approximation is shown below.

(13) $$p\left(x\right)=\frac{\exp\left(-x\right)(x^{3}+18x^{2}+86x+96)}{x\left(x^{4}+20x^{3}+120x^{2}+240x+120\right)}$$

#### 2.3.3. Thermodynamic Analysis

Thermodynamic quantities for pyrolysis and oxidative thermal degradation of tennis string nylon-6 waste, including activation energy (*E*_a_), changes in enthalpy (Δ*H*), changes in entropy (Δ*S*), and changes in Gibbs free energy (Δ*G*), were calculated based on transition-state theory using the following expressions [24,28]:

(14) $$A=\frac{ek_{B}T}{h}\exp(\frac{\Delta S}{R})$$

Δ*H* = *E_a_* − *RT* (15)

Δ*G* = Δ*H* − *T*Δ*S* (16)

where *e* is the Neper number (2.7183), *h* and *k*_B_ represent the Plank constant (6.626 × 10^−34^ J/s) and Boltzmann constant (1.381 × 10^−23^ J/K), respectively. Herein, *A* is the pre-exponential factor determined as above and *E*_a_ the activation energy from the SK method.

## 3. Results and Discussion

### 3.1. FTIR Analysis of Nylon-6 Sample

**Figure 1:**
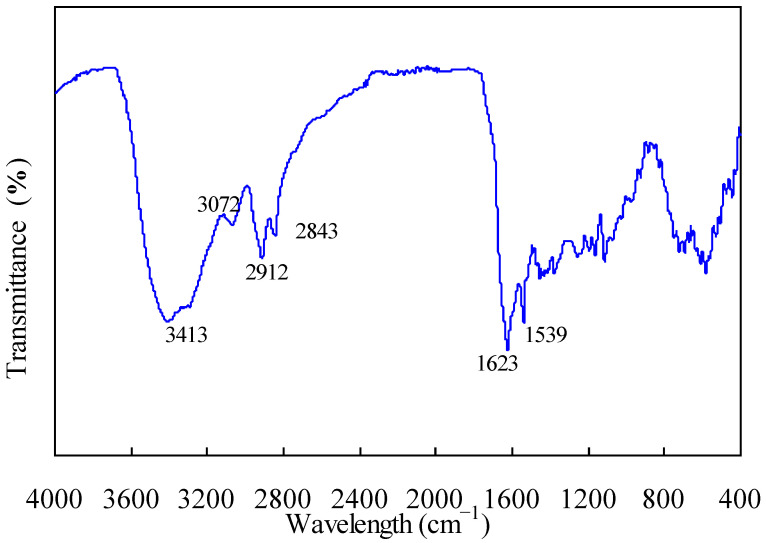
FTIR analysis spectra determined for nylon-6 tennis string waste samples.

Figure 1 shows the FTIR identification of chemical groups involved for nylon-6 tennis filament waste, and these FTIR results for nylon-6 are almost the same as those documented in the literature [29,30]. The absorption band peaked at 3413 cm^−1^ can be attributed to the stretching vibrations of hydrogen-bonded NH groups while the adsorption bands centered at 3072, 2912, and 2843 cm^−1^ can be assigned to the stretching vibration of aliphatic CH groups for the nylon-6 waste sample. Meanwhile, the absorption peak at 1623 cm^−1^ can be accounted for as the stretching vibration of amide II and CN groups of nylon-6 and the one at 1539 cm^−1^ may be due to the vibrations of amide I [30]. The results indicate that the collected filament sample is primarily made of nylon-6 polymer.

### 3.2. Thermal Degradation Characteristics of Nylon String Wastes

**Figure 2:**
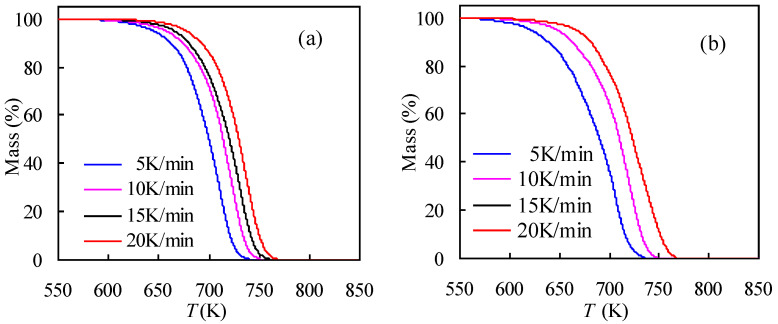

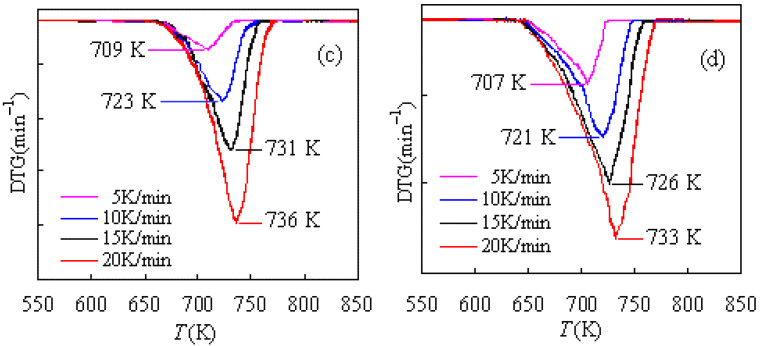
TG (**a**,**b**) and DTG (**c**,**d**) results of tennis string nylon samples measured in the atmosphere of inert N_2_ (**a**,**c**) and oxidative air (**b**,**d**).

Figure 2 presents multi-heating-rate TG curves of tennis nylon-6 string waste samples determined in two different atmospheres of oxidative air and inert N_2_ and programmed from ambient to 850 K under four ramping rates of *β* = 5, 10, 15, and 20 K/min. Clearly, as found from the TG results, the mass-*T* curve has moved to higher temperature ranges upon enlarging the heating rate *β* and the temperature movement is applied to both cases. This result may be attributed to a combined effect of different heat transfer and distinct kinetic rates, which in turn, lead to the reduced gasification rate of the reaction product with the rise in heating rate and subsequently a temperature-shift compensation.

A careful examination of Figure 2 shows that nylon-6 waste retains relatively stronger thermal stability in inert N_2_ than in oxidative air, but in both cases, the waste sample has completely burned out without any visible solid residual. As can be seen, nylon-6 begins to degrade in oxidative air when heated upwards to 581 or 622 K upon employing different heating rates, and its complete degradation may be accomplished when heating the sample up to 737 K under 5 K/min or 770 K for the case of 20 K/min. In contrast, the pyrolysis in inert N_2_ for nylon-6 string wastes is seen to undergo in-between 606 to 742 K or from 643 to 772 K when augmenting *β* from 5 K/min to 20 K/min. With these TG data, kinetics analysis can be performed for inert and oxidative thermal degradation processes and will be discussed later. Similarly, Pannase et al. [11] recently reported that for fresh nylon ropes, major degradation occurred in the temperature range of 673 to 793 K, and such observations can be also found in the other studies [9,10].

Figure 2 also shows DTG results of nylon-6 string wastes obtained under multiple heating rates for both N_2_ and air atmospheres, and one can see that all DTG curves have displayed only one peak for every heating rate, at which the sample has reached a maximum mass loss rate (d*α*/d*t*) and the temperature at the peak is normally defined as *T*_p_. Like those shown for the TG results, the DTG results are also significantly affected by the variation in *β* and DTG curves moving rightwards to high-temperature domains as *β* rises as reflected by Figure 2. Meanwhile, the *T*_p_ value is ascended and the peak turns wide and intense as *β* rises up from 5 to 20 K/min. These observations are the same for both cases of N_2_ and air. As seen from Figure 2, the *T*_p_ values are unsurprisingly lower in air than in N_2_. Furthermore, one can deduce from these one-peak DTG results that the nylon-6 string wastes may have undergone one reaction stage during thermal degradation processes and thus a single-reaction model assumption may be somewhat reasonable for describing kinetic degradations according to ICTAC Kinetics Committee recommendations [13]. Such an assumption is useful for the purpose of later finding the mechanism function for pyrolysis and oxidative thermal degradation of nylon-6 tennis string wastes.

**Figure 3:**
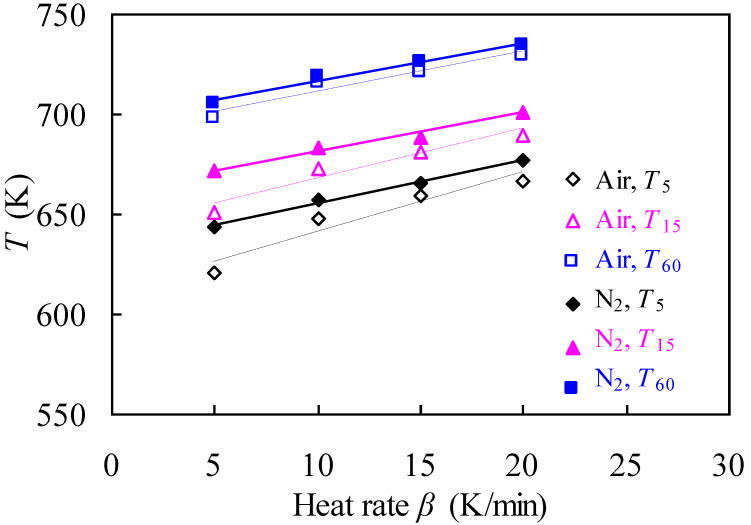
Effect of heating rate *β* on specific degradation temperatures of the nylon-6 string waste sample in oxidative air and inert N_2_.

Looking at Figure 3 where some specific temperatures directly abstracted from TG data are plotted, one can see how the heating rate affects the thermal degradation of nylon-6 string waste. The temperatures randomly selected here include *T*_5_, *T*_15_, and *T*_60_, and they are defined as the temperatures, respectively, at 5% mass conversion (*T*_5_), 15% mass conversion (*T*_15_), and 60% mass conversion (*T*_60_). For both cases of inert and oxidative atmospheres, each of these specific temperatures is linearly promoted with the increase in the heating rate and a linear expression may be derived for quantitatively describing the heating-rate dependence of each specific temperature. As can be seen from Figure 3, under the same heating rate, these specific temperatures are all higher in inert nitrogen than in oxidative air. Similar observations have also been reported in our earlier work where an ionic liquid of [bmim]PF_6_ demonstrated rather better thermal stability in N_2_ than in air [25]. 

**Figure 4:**
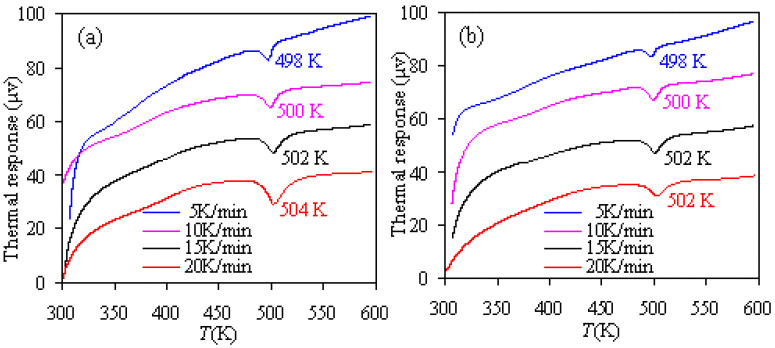
DTA results of nylon-6 tennis string waste measured in N_2_ (**a**) and air (**b**).

During pyrolysis, the nylon-6 tennis string waste sample experienced two heat response stages—endothermic melting and exothermic decomposition, respectively. Figure 4 presents the endothermic melting involved DTA curves under four different hearing rates for the nylon-6 waste. It can be seen that the nylon-6 possesses a crystalline structure and these crystalline properties are strongly impacted by the heating rate as evidenced by the variation in the melting point with the heating rate shown in Figure 4. In N_2_, the crystal in the nylon-5 waste sample melts at 498, 500, 502, and 504 K when heated at 5, 10, 15, and 20 K/min. In the case of air atmosphere, the melting points become relatively lower as compared to the inert case, similar to those TG data discussed above. These melting point values are consistent with those in the literature [31,32] and can be attributed to the presence of a thermodynamically stable α-form crystal of nylon-6, which is formed with hydrogen-bonded chains packed in an anti-parallel manner [31,32].

### 3.3. Kinetics Analysis of Thermal Degradation 

#### 3.3.1. Results Obtained by the SK Method

**Figure 5:**
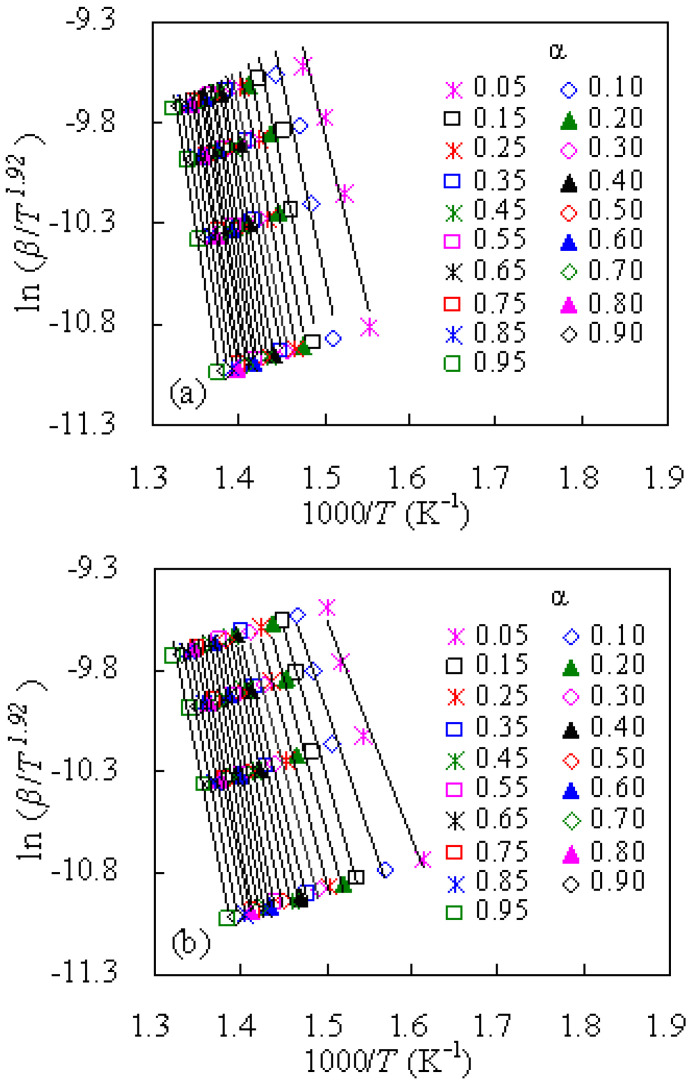
Linear SK plots of ln (*β*/*T*^1.92^) vs. 1000/*T* obtained for tennis nylon waste in inert N_2_ (**a**) and air (**b**).

With the SK method, the plots of ln(*β*/*T*^1.92^) against 1000/*T* for the nylon-6 degradation in both air and N_2_ are presented in Figure 5, while the *α*-dependent *E_a_* values calculated from the slope of each Arrhenius plot are listed in Table 2. The *R*^2^ values are also listed in Table 2, and as can be seen, they are close to 1.00, revealing a linear dependence of ln(*β*/*T^1.92^*) on 1/*T* for each conversion level.

The *E_a_* in N_2_ is found from Table 2 to intensely rely on α and it varies within 142.79–207.97 kJ/mol as α rises up from 0.05 to 0.95, and the averaged *E_a_* value is 188.95 kJ/mol over the whole conversional range. The difference between the largest *E_a_* at α = 0.95 and smallest *E_a_* at α = 0.05 or the second smallest *E_a_* at α = 0.10 is about 34.49% or 22.34% of the averaged *E_a_*. Thus, the disparity between the averaged *E_a_* and *E_a_* of 0.10 < α < 0.95 can be roughly taken constantly over all conversion levels and a one-step degradation model can be approximately assumed. However, the gradual increase in *E*_a_ with α turns to indicate that thermal degradation may become a little more complex with the progress of mass conversion. In terms of the *E*_a_, very similar or higher results have been reported in the literature [4,11,12]. In the study of Holland and Hay [4], an averaged *E*_a_ of 190 kJ/mol, rather close to our work, was obtained for pyrolysis of fresh nylon-6. Likewise, a higher *E*_a_ of 205 kJ/mol was resulted for nylon-6 pyrolysis by means of model-free SK, CR, and FWO methods [11]. Moreover, the averaged *E*_a_ of 205 or 211 kJ/mol was calculated for isothermal or non-isothermal conditions, respectively, for pyrolysis of nylon-6 in helium [12]. Moreover, Kim et al. [5] found that the *E*_a_ of nylon-6 fishing net wastes was considerably lower by 46 kJ/mol than that of fresh net samples, indicative of the possible environmental effect on pyrolysis degradation of nylon-6. From this aspect, one may deduce from relatively lower *E*_a_ values that the nylon-6 tennis string waste, investigated here, may have experienced mechanical degradation upon stroking high-speed shots during tennis.

On the other hand, the *E_a_* values for oxidative thermal degradation are relatively lower than those for the N_2_ case, and these values span from 90.45 to 178.16 kJ/mol. Such a result tends to suggest that the chemical reaction of varying degrees of complexity might be involved during oxidative degradation. The gap between the α-dependent *E_a_* and the averaged *E_a_* is relatively larger as compared to the inert N_2_ case. However, for the purpose of comparing two different thermal gases the one-step degradation model assumption has been taken. In this way, the model-fitting methodology with kinetic SK method and the other methods such as Tang, MKN, CR, or FWO methods shown later may be plausible for elaborating kinetic thermal degradation according to the ICTAC Kinetics Committee suggestions [13].

#### 3.3.2. Results Obtained by the Tang Method

The isoconversional Tang method is considered for analyzing thermal degradation kinetics of string wastes in both N_2_ and air atmospheres. According to the Tang method, the Arrhenius plots of ln(*β*/*T*^1.896441^) vs. 1000/*T* over 0.05 < *α* < 0.95 are given in Figure S1 for oxidative thermal degradation and N_2_ pyrolysis processes. Similar to the SK method, a very good linear relationship can be observed between ln(*β*/*T*^1.896441^) and 1/*T,* and the *R*^2^ values can be referred to in Table S1. From the slope of each Arrhenius line, the *E_a_* values can be estimated using Equation (7) and the calculated results for all *α* values are presented in Table S1. Compared to the *E_a_* data in Table 2, one can see that the *E_a_* value computed by using the Tang method shows the same trend as that resulted from the SK method. For the case of oxidative degradation, the activation energy *E_a_* value shows relatively large deviations and it varies from 90.53 to 178.20 kJ/mol for 0.05 < *α* < 0.95. In contrast, the *E_a_* value for N_2_ pyrolysis varies within from 142.84–207.99 kJ/mol. The averaged *E_a_* values are 151.09 and 188.98 kJmol^−1^ for air and N_2_ cases, respectively. Unsurprisingly, the deviation of the α-dependent *E_a_* from the averaged *E_a_* for both air and N_2_ cases exhibited a similar result to that observed for the SK method. Hence, the Tang method could be used for describing pyrolysis in N_2_ or oxidative thermal degradation regardless of relatively large activation energy fluctuations.

#### 3.3.3. Results Obtained by the MKN Method

Similar to the SK and Tang methods, the isoconversional MKN method has been employed to determine the *E_a_* value following Equation (8). Accordingly, the Arrhenius plots of ln(*β*/*T*^1.884318^) vs. 1000/*T* over 0.05 < *α* < 0.95 are also shown in Figure S2 and the *E_a_* values thus obtained are tabulated in Table S2 for the nylon-6 waste sample. These results show that the *E_a_* values alter with the conversional extent in N_2_ with a relatively larger deviation than in air. In the case of N_2_, the *E_a_* value gains momentum from 142.82 to 188.95 kJ/mol as α increases and it varies within 90.54–178.17 kJ/mol for oxidative thermal degradation. The averaged *E_a_* over the entire mass loss range is 188.95 for N_2_ and 151.08 for air. 

#### 3.3.4. Results Obtained by the CR Method

Attempting the CR method for analyzing kinetics, the Arrhenius plots of ln(*β*/*T^2^*) versus 1000/*T* over 0.05 < α < 0.95 for thermal degradation in air or N_2_ pyrolysis processes are presented in Figure S3, and the *E_a_* calculated from the slope of each plotted line according to Equation (9) and resultant *R*^2^ values are given in Table S3. As can be observed from Figure S3, the linear relationship between ln(*β*/*T^2^*) and 1/*T* is very well as is also reflected by the *R*^2^ values listed in Table S3. As also shown in Table S3, the *E_a_* value relies intensely on mass conversion α and it spans from 90.10 to 177.83 kJ/mol for N_2_ pyrolysis while from 142.46 to 207.64 kJ/mol for oxidative thermal degradation. On average, the *E_a_* values thus obtained are 150.69 and 188.63 kJ/mol for air and N_2_ cases, respectively. 

#### 3.3.5. Results Obtained by the FWO Method

Following the FWO method, the Arrhenius plots of log(*β*) against 1000/*T* are depicted in Figure S4 as well for the nylon-6 waste sample in both air and N_2_, and the *E_a_* and *R*^2^ values thus calculated for each *α* are given in Table S4. The *R*^2^ values over the entire conversion range are found to closely approximate 1.00, showing a linear dependence of log(*β*) on 1/*T* for every conversion level. The *E_a_* values via the FWO method are known to be readily estimated from the line slopes of all Arrhenius plots shown in Figure S4. From Table S4, one can see that the *E_a_* values for either N_2_ or air are strongly dependent on α and they vary from 95.82 to 180.42 kJ/mol for pyrolysis or from 145.91 to 209.15 kJ/mol for oxidative thermal degradation as α varies from 0.05–0.95. If averaged over the entire conversion range, the *E_a_* values thus calculated are 190.61 and 154.38 kJ/mol, respectively. 

A comparison made among all five integral methods shows that both SK and CR methods have almost the same conversion-dependent *E_a_* curves, and the SK method led to 0.32 kJ/mol higher *E_a_* values in N_2_ than the CR method or 0.35 kJ/mol higher in air at all conversional levels. As for the other three methods, they all resulted in very similar *E_a_* versus α curves to the SK and CR methods. Of all five methods, the *E_a_* values at all α values increased, following the sequence: CR < SK < MKN < Tang < FWO for both cases of N_2_ and air atmospheres. A further examination shows that for the inert N_2_ pyrolysis process, the *E_a_* values calculated from the CR method are 0.33, 0.35, and 1.98 kJ/mol lower on average than those, respectively, from the MKN, Tang, and FWO methods, while in the case of oxidative thermal degradation, the *E_a_* values from the CR method are on average 0.38, 0.39, and 3.69 kJ/mol smaller than those from the MKN, Tang, and FWO methods, respectively. In other words, the SK, CR, MKN, and Tang methods have generally comparable calculation performances even though they involve different approximations. The FWO method seems to always give relatively large *E_a_* results [23,24,25]. 

### 3.4. Model-Fitting Analysis

**Figure 6:**
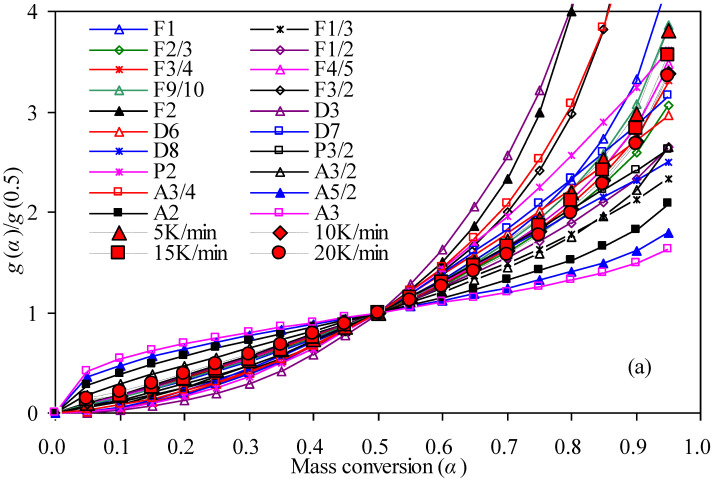

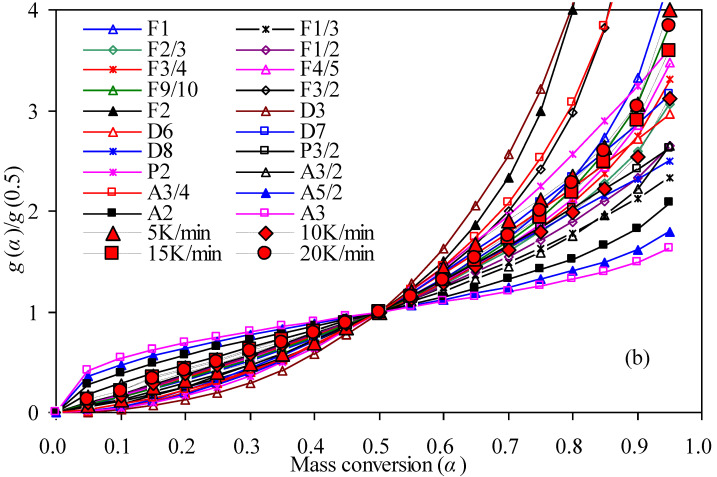
Theoretical and experimental G-master plots for nylon-6 string waste samples: (**a**) in N_2_ and (**b**) in air.

Apart from obtaining the *α*-dependent *E*_a_ from TG data with five model-free methods, the kinetic model *f* (*α*) of nylon-6 waste has been identified by using the model fitting method. Thus, the G-master plots method has been applied here for this purpose [11,21,22,26] and Figure 6 shows theoretical master plots of *g*(*α*)/*g*(0.5) vs. *α* against experimental plots of 5, 10, 15, and 20 K/min for both air and N_2_ cases. All the reaction functions presented in Table 1 are considered here to result in a number of theoretical master plots based on Equation (12). It may be noted that among these reaction models considered, four types of chemical reaction order equation, acceleratory nucleation equation, sigmoidal Avrami–Erofeev equation, and deceleratory three-dimensional diffusion equation have been compared against experimental curves by their matching extents. For the N_2_ case, only seven chemical reaction equations of F1/3, F1/2, F2/3, F3/4, F4/5, F9/10, and F1 have given rather better condensations on experimental plots for 0.05 < *α* < 0.50 whereas the other reaction models are observed to be further away from the experimental values. While for 0.50 < *α* < 0.95, five Avrami–Erofeev mechanism functions, D3, D7, F2, F3/2, F1/3, and P2 models are observed to lead to very large deviations against the experimental data, and D6, D8, F1/2, and P3/2 models are seen to result in certain deviations as compared to experimental values, the remaining five chemical order mechanism functions of F2/3, F3/4, F4/5, F9/10, and F1 are found to very well condense on experimental plots. 

In the case of oxidative thermal degradation, the same finding for the N_2_ case is applied as *α* varies in the range of 0.05–0.50 as shown in Figure 6. On the other hand, the fittings seem relatively different for 0. 50 < *α* <0.95, the Avrami–Erofeev mechanism functions including A3/4, A3/2, A2, A5/2, and A3, along with D3, P2, F3/2, F2, F1/2, and F1/3 models, resulted in very large deviations against experimental curves while P3/2, D6, D7, and D8 models also led to minorly poor performance. Promisingly, the left F2/3, F3/4, F4/5, F9/10, and F1 reaction models are seen to very well approximate experimental plots. Due to the assumption of the one-stage reaction for the entire oxidative and pyrolysis thermal degradation processes, the F2/3, F3/4, F4/5, F9/10, and F1 reaction models can then be considered further as they perform very well over 0.05 < *α* <0.95, and their simulated results will be compared so as to identify the most suitable reaction models for thermal degradation of nylon-6 string waste. In the literature, the first-order reaction function for pyrolysis of nylon-6 and its waste has already been reported [4,5,7], while a degradation mechanism model with a chemical reaction order of 0.81–1.9 has been documented for catalytic pyrolysis of nylon-6 by Bockhorn et al. [12]. Furthermore, the work of Pannase et al. [11] showed a very different contracting sphere R3 model other than the chemical order model for pyrolysis degradation of nylon-6.

**Figure 7:**
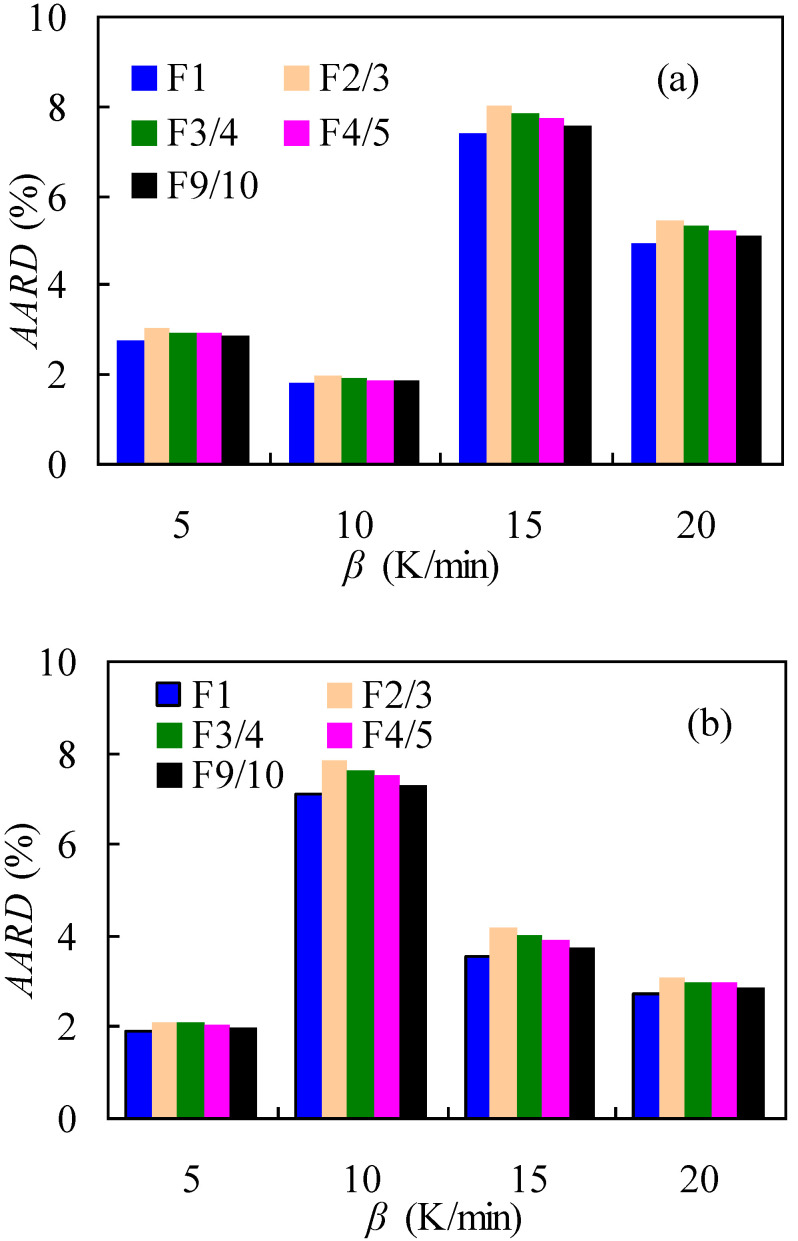
The AARD results under different *β* values for some reaction models with the CR method: (**a**) in nitrogen and (**b**) in air.

The comparison of the above-mentioned five models was conducted by fitting them against experimental data by the very commonly CR method in terms of the absolute average relative deviation (AARD) defined as:

(17) $$AARD(\%)=\sum_{i=1}^{N}\left(\frac{\left|\alpha_{\exp}-\alpha_{cal}\right|}{\alpha_{\exp}}\right)/N\cdot 100$$

where *α_exp_* and *a_cal_* are, respectively, the mass conversion experimentally obtained and calculated with the reaction model, and *N* is the total number of mass conversion considered here. Figure 7 presents the AARD results from five reaction models, and it can be seen that the F1 model led to the lowest AARD values among the five models for both air and N_2_ cases. In this sense, the F1 model can be readily thought of as the most appropriate reaction model for pyrolysis and oxidative thermal degradation of nylon-6 tennis string wastes. With the *E*_a_ and F1 model, the pre-exponential factor ln*A* values were then calculated according to Equation (8) and the results thus obtained—as a function of *α*—are listed in Table 3 for both inert and oxidative atmospheres. The calculated ln*A* results presented in Table 3 show that the ln*A* value varies from 12.50 to 29.03 for the air case, and from 22.13 to 34.11 for the N_2_ case, respectively. Accordingly, the *A* value ranges from 2.68 × 10^5^ to 4.03 × 10^12^ min^−1^ and 4.08 × 10^9^ to 6.51 × 10^14^ min^−1^ for the air and N_2_ cases, respectively.

**Figure 8:**
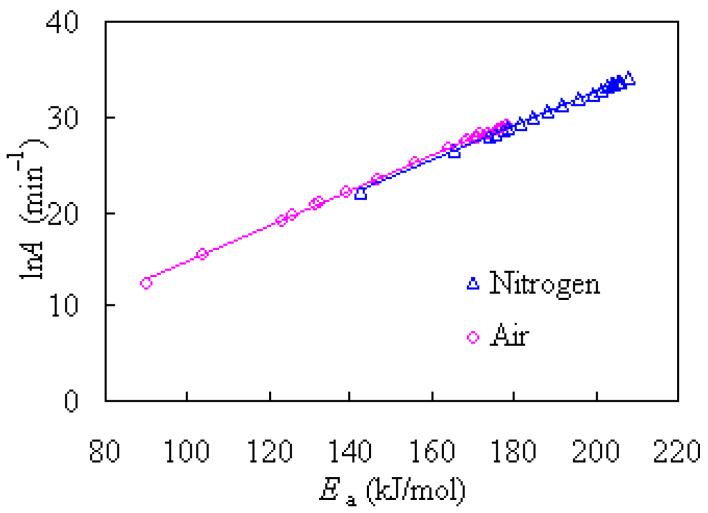
Compensation effect observed between ln*A* and *E_a_* for nylon-6 tennis string waste.

A further examination shows that there is a kinetic compensation effect between the *E*_a_ and ln*A*, indicative of a compensatory increase in ln*A* with an increase in *E_a_* [23]. Such a compensation effect is graphically presented in Figure 8 and can be linearly expressed as the following:

N_2_: ln*A* = 0.1818 *E_a_* − 3.6882 (*R*^2^ = 0.9998)

Air: ln*A* = 0.1855 *E_a_* − 3.7944 (*R*^2^ = 0.9990)

where the units of *E*_a_ and ln*A* are kJ/mol and min^−1^, respectively.

**Figure 9:**
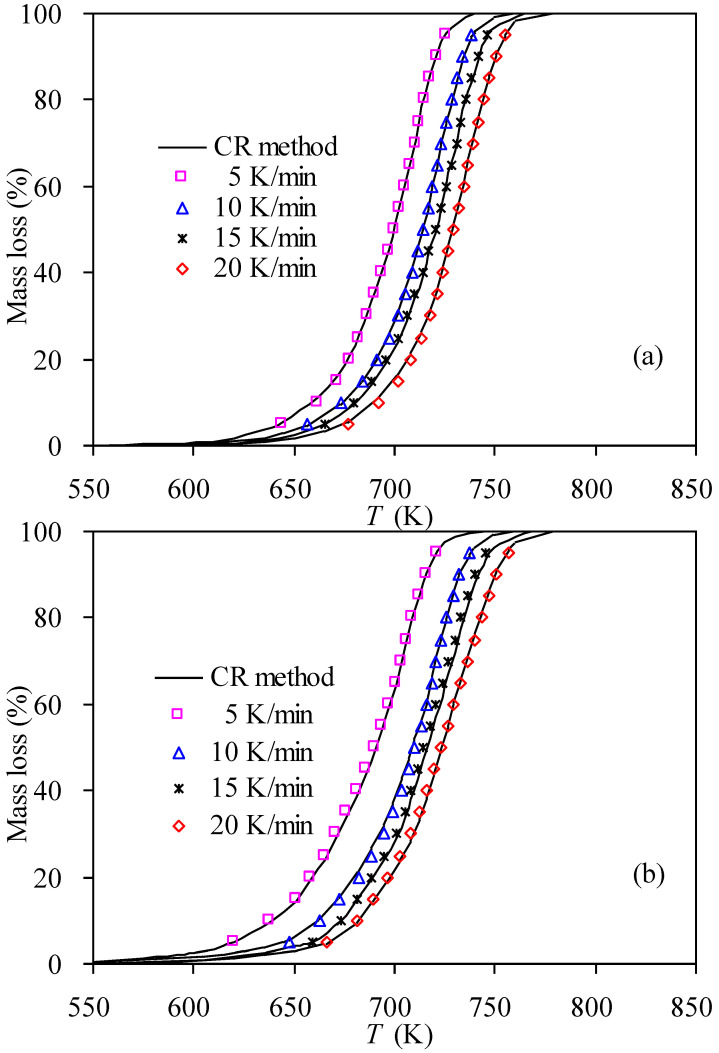
Recast α ~ T curves of for thermal degradation of nylon-6 wastes in (**a**) N_2_ and (**b**) air.

With fully obtained kinetic parameters, the theoretical predictions to thermal degradation processes can be made, and Figure 9 presents the matching results against experimental values using the CR method. It can be observed that the F1 model coupled with the *E*_a_ and ln*A* parameters over the entire range of 0.05–0.95 performed satisfactorily to recast the temperature-dependent mass-conversion curves for nylon-6 waste samples since all the experimental data points selected are mostly condensed on their respective *α*-*T* curves. 

### 3.5. Thermodynamic Analysis

Thermodynamic analysis of pyrolysis and oxidative thermal degradation processes for nylon-6 tennis string wastes can be accomplished based on the assumption of transition state theory [11,24,28]. Accordingly, thermodynamic parameters in terms of Δ*G*, Δ*H*, and Δ*S* have been evaluated with the aid of the *E_a_* values resulting from the CR method and ln*A* from the F1 model, and the calculation results are listed in Table 3. As can be seen, nylon-6 tennis string waste has a lower Δ*H* value in oxidative air than in nitrogen over the entire conversion range, requiring less energy to degrade at any conversion level. All the Δ*H* values are positive in either air or N_2_, and such endothermic features mean oxidative and inert thermal degradations are all heat absorbed. As seen from Table 3, the Δ*G* values are almost kept constant at 186.51 and 185.85 kJ/mol over 0.05 < *α* < 0.95 for nitrogen and air cases, respectively. Thermodynamically, all positive Δ*G* means non-spontaneous degradation processes in both atmospheres, and therefore, heat must be introduced to undergo thermal degradation. The higher the Δ*G*, the less favorable to undertake the degradation process. Finally, most Δ*S* values are negative during the degradation process as observed from Table 3, confirming the production of a more structurally ordered activated complex based on the transition state theory. However, such a transition from a disordered state to an ordered state is questionable since polymer macromolecular chains will certainly collapse into small molecules during high-temperature degradation. Further investigation should be conducted to deeply comprehend the thermodynamics of thermal degradation processes. Overall, the Δ*H* and Δ*G* values span from 84.71–171.85 kJ/mol and 185.40–186.41 kJ/mol for oxidative degradation, and 136.97–201.48 kJ/mol and 184.59–187.44 kJ/mol for N_2_ pyrolysis, respectively. Meanwhile, the Δ*S* values vary from −75.85 to 22.79 J/mol·K for pyrolysis or from −155.76 to −19.23 J/mol·K for oxidative thermal degradation. Similarly, Pannase et al. [11] also reported thermodynamic quantities for nylon-6 pyrolysis and Δ*H*, Δ*G*, and Δ*S* values—198.89 kJ/mol, 199.72 kJ/mol, and −1.09 J/mol·K, respectively.

## 4. Conclusions

In the present study, non-isothermal features and degradation kinetics of nylon-6 tennis string waste were studied under N_2_ and oxidative air atmospheres with TG measurements at a ramping rate of 5–20 K/min. Five integral methods—SK, Tang, MKN, CR, and FWO—were carried out to calculate activation energies over the entire conversion range. The master-plot method was used to search the most appropriate degradation model for describing pyrolysis and oxidative thermal degradation of nylon-6 waste. Some concluding remarks may be drawn from the present work:

(1) Nylon-6 waste is more thermally stable in N_2_ than in air, meanwhile, the degradation thermal temperatures were found to linearly rise with the heating rate.
(2) For kinetically analyzing thermal degradation of nylon-6 waste, a one-stage reaction model is assumed for elaborating both non-isothermal thermo-oxidative degradation and N_2_ pyrolysis.
(3) The *E_a_* values over 0.05 < α < 0.95, calculated by using the isoconversional SK method, vary from 95.82 to 180.42 kJ/mol for N_2_ pyrolysis or from 145.91 to 209.15 kJ/mol for oxidative thermal degradation, respectively. The variation in *E_a_* with respect to α suggests that the chemical reaction of varying extents of complexity may have occurred during pyrolysis or oxidative degradation.
(4) Among five model-free methods, the FWO method yielded the highest *E_a_* values while the lowest *E_a_* values were obtained by using the CR method, and the *E_a_* value over the entire conversion range follows the sequence: CR < SK < MKN < Tang < FWO for both atmospheres.
(5) By means of the model-fitting G-master-plots method, many reaction models were evaluated with the most common CR method and the first-order reaction model was found to be the most suitable mechanism function for describing pyrolysis and oxidative degradation of nylon-6 waste—as proven by the lowest calculation deviations. The CR method together with kinetic triplet parameters rebuilt multiple α-T curves with satisfactory performances.
(6) Thermodynamically, Δ*H*, Δ*S*, and Δ*G* were estimated according to the transition state theory, and over the entire degradation process, the Δ*H*, Δ*G*, and Δ*S* values thus calculated varied from 137 to 201 kJ/mol, 185 to 187 kJ/mol, and −76 to 23 J/mol·K for N_2_ pyrolysis, or from 85 to 172 kJ/mol, 185 to 186 kJ/mol, and −156 to −19 J/mol·K for oxidative degradation, respectively. These results suggest that it is unfavorable for nylon-6 tennis string wastes to undergo thermal degradation and more energy is required for conducting pyrolysis than utilizing oxidative thermal degradation.

## Figures and Tables

**Table 1:** Selected reaction models used for describing thermal degradation of nylon-6 tennis string waste.

Model	*g*(α)	*f*(α)	Rate-Determining Mechanism
Chemical process equation			
F1/3	1-(1 − α)^2/3^	(3/2)(1 − α)^1/3^	Chemical reaction
F1/2	1-(1 − α)^1/2^	2(1 − α)^1/2^	
F2/3	1-(1 − α)^1/3^	3(1 − α)^2/3^	
F3/4	1-(1 − α)^1/4^	4 (1 − α)^3/4^	
F4/5	1-(1 − α)^1/5^	5 (1 − α)^4/5^	
F9/10	1-(1 − α)^1/10^	10 (1 − α)^9/10^	
F1	−ln(1 − α)	1 − α	
F3/2	(1 − α)^−1/2^ − 1	2(1 − α)^3/2^	
F2	(1 − α)^−1^ − 1	(1 − α)^2^	
Fn (*n* ≠ 1)	[(1 − α)^1-n^ − 1]/(n − 1)	(1 − α)^n^	
Acceleratory rate equation			
P3/2	α^3/2^	(2/3)α^−1/2^	Nucleation (power law)
P2	α^2^	(1/2)α^−1^	
Sigmoidal rate equation			
A3/4	[−ln(1 − α) ]^4/3^	(3/4)(1 − α)[−ln(1 − α)]^−1/3^	Random nucleation (Avrami–Erofeev)
A3/2	[−ln(1 − α) ]^2/3^	(3/2)(1 − α)[−ln(1 − α)]^1/3^	
A2	[−ln(1 − α) ]^1/2^	2(1 − α)[−ln(1 − α)]^1/2^	
A5/2	[−ln(1 − α) ]^2/5^	(5/2)(1 − α)[−ln(1 − α)]^3/5^	
A3	[−ln(1 − α)]^1/3^	3(1 − α)[−ln(1 − α)]^2/3^	
An (*n* ≠ 1)	[−ln(1 − α) ]^1/n^	n(1 − α)[−ln(1 − α)]^(1−1/n)^	
Deceleratory rate equation			
D3	[1 − (1 − α)^1/3^]^2^	(3/2)(1 − α)^2/3^[1 − (1 − α)^1/3^]^−1^	Three-dimensional diffusion
D6	[(1 + α)^1/3^ − 1]^2^	(3/2)(1 + α)^2/3^[(1 + α)^1/3^ − 1]^−1^	
D7	1 + (2/3)α − (1 + α)^2/3^	(3/2)[(1 + α)^−1/3^ − 1]^−1^	
D8	[(1 + α)^−1/3^ − 1]^2^	(3/2)(1 + α)^4/3^[(1 + α)^−1/3^ − 1]^−1^	

**Table 2:** Activation energies calculated using the SK method for thermal degradation of nylon-6 tennis string wastes.

α	N_2_		Air	
	*E_a_* (kJ/mol)	*R* ^2^	*E_a_* (kJ/mol)	*R* ^2^
0.05	142.79	0.9697	90.45	0.9818
0.10	165.74	0.9430	104.37	0.9901
0.15	174.02	0.9419	123.21	0.9917
0.20	175.52	0.9541	126.07	0.9732
0.25	177.48	0.9662	131.51	0.9713
0.30	178.96	0.9670	132.43	0.9688
0.35	181.75	0.9714	139.18	0.9681
0.40	184.68	0.9793	146.79	0.9641
0.45	188.37	0.9845	156.03	0.9664
0.50	192.12	0.9878	164.13	0.9715
0.55	195.74	0.9900	171.30	0.9770
0.60	199.45	0.9910	176.24	0.9819
0.65	201.73	0.9921	178.16	0.9851
0.70	203.08	0.9924	177.28	0.9870
0.75	204.21	0.9928	173.68	0.9880
0.80	204.81	0.9925	170.48	0.9888
0.85	205.73	0.9923	168.62	0.9898
0.90	205.94	0.9912	168.00	0.9908
0.95	207.97	0.9901	171.80	0.9860
Average	188.95 ± 16.73		151.04 ± 25.87	

**Table 3:** Thermodynamic parameters of Δ*H*, Δ*S,* and Δ*G* calculated for thermal degradation of nylon-6 tennis string wastes.

α	N_2_				Air			
	lnA	ΔH	ΔS	ΔG	lnA	ΔH	ΔS	ΔG
	(min^−1^)	(kJ/mol)	(J/mol K)	(kJ/mol)	(min^−1^)	(kJ/mol)	(J/mol K)	(kJ/mol)
0.05	22.13	136.97	−75.85	187.10	12.50	84.71	−155.76	185.72
0.10	26.42	159.80	−40.37	187.12	15.45	98.50	−131.41	185.74
0.15	27.92	167.99	−28.06	187.26	19.11	117.27	−101.14	185.41
0.20	28.19	169.44	−25.90	187.40	19.69	120.06	−96.40	185.74
0.25	28.54	171.35	−23.04	187.44	20.70	125.44	−88.09	186.04
0.30	28.81	172.79	−20.80	187.41	20.88	126.31	−86.66	186.41
0.35	29.32	175.55	−16.64	187.31	22.11	133.03	−76.45	186.41
0.40	29.84	178.45	−12.32	187.20	23.49	140.60	−65.05	186.29
0.45	30.50	182.12	−6.91	187.05	25.14	149.82	−51.37	186.09
0.50	31.16	185.85	−1.41	186.86	26.58	157.89	−39.47	185.89
0.55	31.81	189.45	3.89	186.65	27.83	165.04	−29.06	185.75
0.60	32.46	193.13	9.29	186.43	28.69	169.96	−21.94	185.67
0.65	32.87	195.40	12.69	186.22	29.03	171.85	−19.23	185.68
0.70	33.12	196.72	14.75	186.02	28.88	170.95	−20.47	185.73
0.75	33.34	197.84	16.54	185.79	28.27	167.32	−25.53	185.83
0.80	33.47	198.42	17.59	185.56	27.75	164.08	−29.95	185.88
0.85	33.66	199.31	19.12	185.29	27.46	162.19	−32.36	185.85
0.90	33.73	199.49	19.67	185.00	27.40	161.54	−32.92	185.73
0.95	34.11	201.48	22.79	184.59	28.10	165.30	−27.17	185.40

## Data Availability

Here, we declare that the data presented in this study are available on request from the corresponding author.

## Supplementary Materials

The following are available online at https://www.mdpi.com/article/10.3390/ma14247564/s1, Figure S1: Linear Tang plots of ln (*β*/*T* ^1.884318^) vs. 1000/*T* obtained for tennis nylon waste in inert N_2_ (a) and air (b), Figure S2: Linear MKM plots of ln (*β*/*T*^1.884318^) vs. 1000/*T* obtained for tennis nylon waste in inert N_2_ (a) and air (b), Figure S3: Linear CR plots of ln (*β*/*T* ^2^) vs. 1000/*T* obtained for tennis nylon waste in inert N_2_ (a) and air (b), Figure S4: Linear FWO plots of log*β* vs. 1000/*T* obtained for tennis nylon waste in inert N_2_ (a) and air (b), Table S1: Activation energies calculated using kinetic Tang method for nylon-6 string wastes thermal degradation, Table S2: Activation energies calculated using kinetic MKN method for nylon-6 string wastes thermal degradation, Table S3: Activation energies calculated using kinetic CR method for nylon-6 string wastes thermal degradation, Table S4: Activation energies calculated using kinetic FWO method for nylon-6 string wastes thermal degradation.
