# Supplementary material for: Kinetic Analysis of Pyrolysis and Thermo-Oxidative Decomposition of Tennis String Nylon Wastes
Source: Materials (Basel). 2021 Dec 9;14(24):7564. doi: 10.3390/ma14247564 (PMC8704089; doi:10.3390/ma14247564)
Supplement: Supplementary file 1 [file materials-14-07564-s001.zip › materials-1480652-Supplementary.pdf]

materials

MDPI

Article

Kinetic Analysis of Pyrolysis and Thermo-Oxidative Decomposition of Tennis String Nylon Wastes

Haibo Wan [1] and Zhen Huang [2,*]

1 Department of Physical Education, Tianjin University of Commerce, Tianjin 300134, China; wanhaibo@tjcu.edu.cn
2 Department of Packaging Engineering, Tianjin University of Commerce, Tianjin 300134, China
* Correspondence: huangzhen@tjcu.edu.cn; Tel.: +86-22-26686264; Fax: +86-22-26686251

Citation: Wan, H.; Huang, Z. Kinetic Analysis of Pyrolysis and Thermo-Oxidative Decomposition of Tennis String Nylon Wastes. *Materials* **2021**, *14*, 7564. https://doi.org/ 10.3390/10.3390/ma14247564

Academic Editor: Halina Kaczmarek

Received: 10 November 2021
Accepted: 5 December 2021
Published: 9 December 2021

Publisher's Note: MDPI stays neutral with regard to jurisdictional claims in published maps and institutional affiliations.

Copyright: © 2021 by the authors. Submitted for possible open access publication under the terms and conditions of the Creative Commons Attribution (CC BY) license (https://creativecommons.org/licenses/by/4.0/).

Table S1. Activation energies calculated using kinetic Tang method for nylon-6 string wastes thermal degradation.

α.	N_2		Air	
	E_a(kJ/mol)	R^2	E_a(kJ/mol)	R^2
0.05	142.84	0.9697	90.53	0.9818
0.10	165.78	0.9431	104.45	0.9901
0.15	174.05	0.9420	123.27	0.9917
0.20	175.56	0.9542	126.13	0.9732
0.25	177.51	0.9663	131.56	0.9713
0.30	178.99	0.9689	132.49	0.9689
0.35	181.78	0.9714	139.24	0.9682
0.40	184.71	0.9794	146.84	0.9642
0.45	188.40	0.9846	156.07	0.9664
0.50	192.15	0.9878	164.17	0.9715
0.55	195.77	0.9901	171.34	0.9770
0.60	199.47	0.9910	176.28	0.9819
0.65	201.76	0.9921	178.20	0.9851
0.70	203.10	0.9924	177.32	0.9870
0.75	204.23	0.9928	173.72	0.9880
0.80	204.83	0.9925	170.52	0.9888
0.85	205.75	0.9923	168.66	0.9898
0.90	205.96	0.9912	168.05	0.9908
0.95	207.99	0.9901	171.85	0.9861
average	188.98±16.73		151.09±25.86	

Table S2. Activation energies calculated using kinetic MKN method for nylon-6 string wastes thermal degradation.

α	N_2		Air	
	E_a(kJ/mol)	R^2	E_a(kJ/mol)	R^2
0.05	142.82	0.9697	90.54	0.9818
0.10	165.76	0.9432	104.45	0.9902
0.15	174.02	0.9420	123.27	0.9917
0.20	175.53	0.9542	126.13	0.9733
0.25	177.48	0.9663	131.56	0.9714
0.30	178.96	0.9670	132.48	0.9689
0.35	181.76	0.9714	139.23	0.9682
0.40	184.68	0.9794	146.83	0.9642
0.45	188.37	0.9846	156.06	0.9664
0.50	192.12	0.9879	164.15	0.9715
0.55	195.74	0.9901	171.32	0.9770
0.60	199.43	0.9910	176.25	0.9819
0.65	201.72	0.9921	178.17	0.9851
0.70	203.06	0.9924	177.30	0.9870
0.75	204.20	0.9928	173.70	0.9880
0.80	204.80	0.9925	170.50	0.9888
0.85	205.71	0.9923	168.64	0.9898
0.90	205.92	0.9912	168.03	0.9908
0.95	207.95	0.9901	171.83	0.9861
average	188.95±16.72		151.08±25.85	

Table S3. Activation energies calculated using kinetic CR method for nylon-6 string wastes thermal degradation.

α	N_2		Air	
	E_a (kJ/mol)	R^2	E_a (kJ/mol)	R^2
0.05	142.46	0.9695	90.10	0.9816
0.10	165.42	0.9427	104.02	0.9901
0.15	173.70	0.9416	122.87	0.9916
0.20	175.20	0.9539	125.72	0.9730
0.25	177.15	0.9660	131.16	0.9711
0.30	178.63	0.9668	132.08	0.9686
0.35	181.43	0.9712	138.84	0.9679
0.40	184.35	0.9792	146.44	0.9639
0.45	188.05	0.9845	155.69	0.9662
0.50	191.80	0.9878	163.79	0.9713
0.55	195.42	0.9900	170.97	0.9768
0.60	199.13	0.9910	175.91	0.9818
0.65	201.42	0.9920	177.83	0.9850
0.70	202.76	0.9923	176.95	0.9869
0.75	203.89	0.9927	173.34	0.9879
0.80	204.49	0.9924	170.13	0.9887
0.85	205.41	0.9923	168.27	0.9897
0.90	205.61	0.9912	167.65	0.9907
0.95	207.64	0.9901	171.45	0.9860
average	188.63±16.71		150.69±25.88	

Table S4. Activation energies calculated using kinetic FWO method for nylon-6 string wastes thermal degradation.

α	N_2		Air	
	E_a (kJ/mol)	R^2	E_a (kJ/mol)	R^2
0.05	145.91	0.9737	95.82	0.9850
0.10	168.01	0.9496	109.33	0.9917
0.15	176.03	0.9484	127.42	0.9928
0.20	177.56	0.9593	130.24	0.9770
0.25	179.49	0.9700	135.51	0.9753
0.30	180.96	0.9707	136.47	0.9732
0.35	183.67	0.9746	142.98	0.9724
0.40	186.51	0.9817	150.28	0.9688
0.45	190.07	0.9863	159.14	0.9705
0.50	193.68	0.9892	166.90	0.9749
0.55	197.17	0.9911	173.79	0.9796
0.60	200.73	0.9920	178.54	0.9839
0.65	202.95	0.9929	180.42	0.9868
0.70	204.26	0.9932	179.64	0.9885
0.75	205.38	0.9936	176.26	0.9895
0.80	205.99	0.9933	173.25	0.9902
0.85	206.90	0.9932	171.53	0.9911
0.90	207.15	0.9922	171.01	0.9920
0.95	209.15	0.9912	174.71	0.9878
average	190.61±16.46		154.38±24.99	

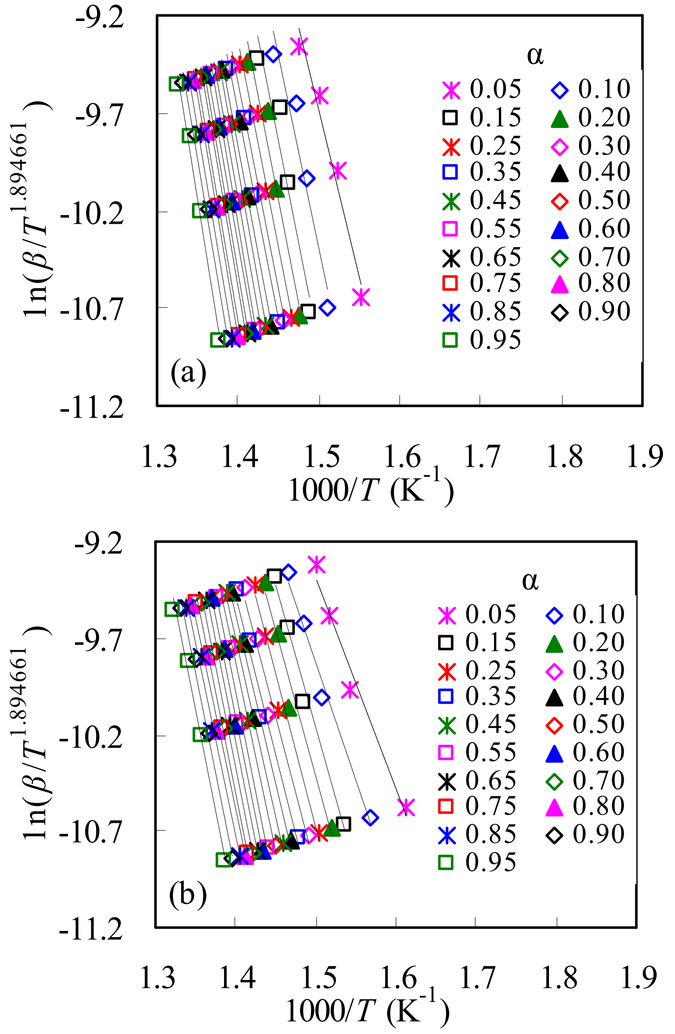

Figure S1. Linear Tang plots of ln ($\beta/T^{1.884318}$) vs. 1000/*T* obtained for tennis nylon waste in inert N_2 (**a**) and air (**b**).

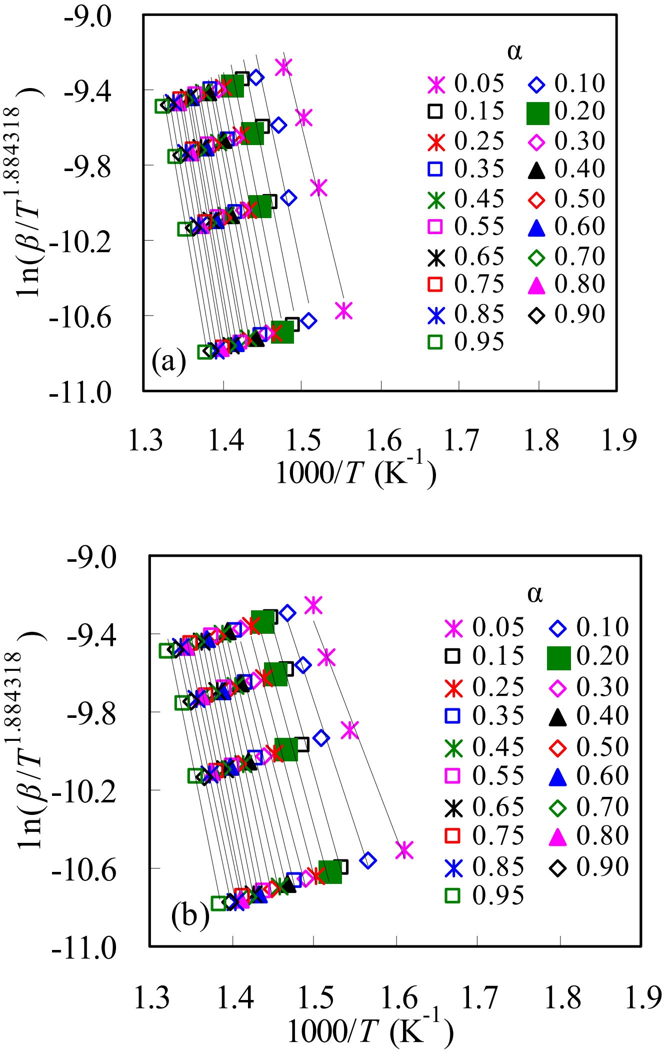

Figure S2. Linear MKM plots of ln ($\beta/T^{1.884318}$) vs. 1000/T obtained for tennis nylon waste in inert N_2 (**a**) and air (**b**).

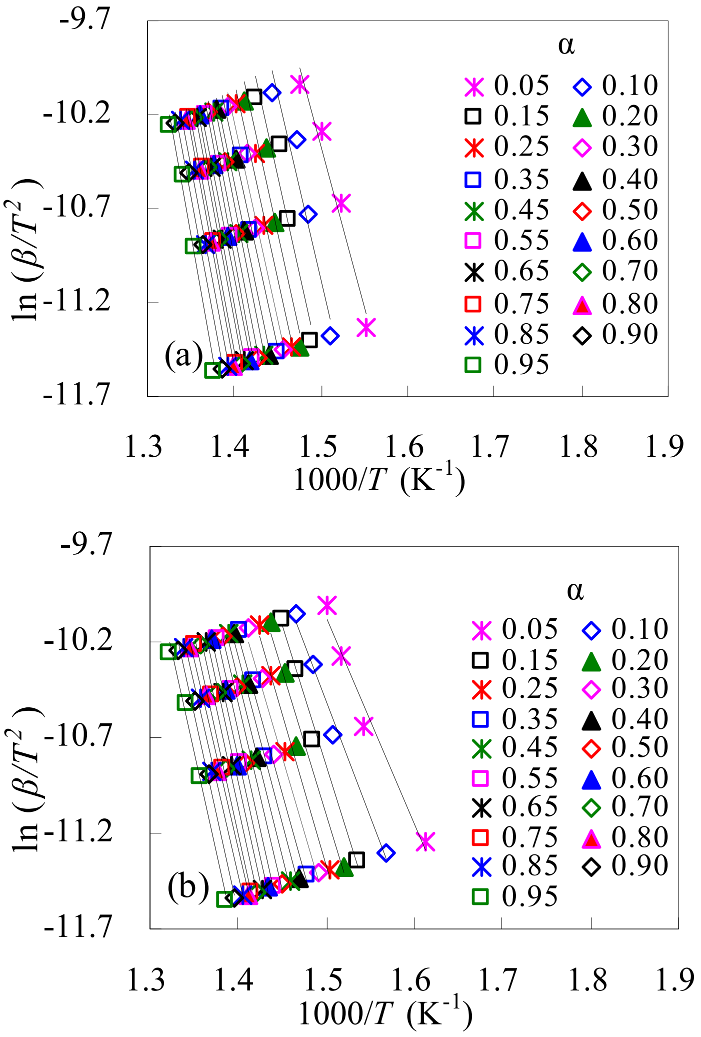

Figure S3. Linear CR plots of ln (β/T^2) vs. 1000/T obtained for tennis nylon waste in inert N_2 (**a**) and air (**b**).

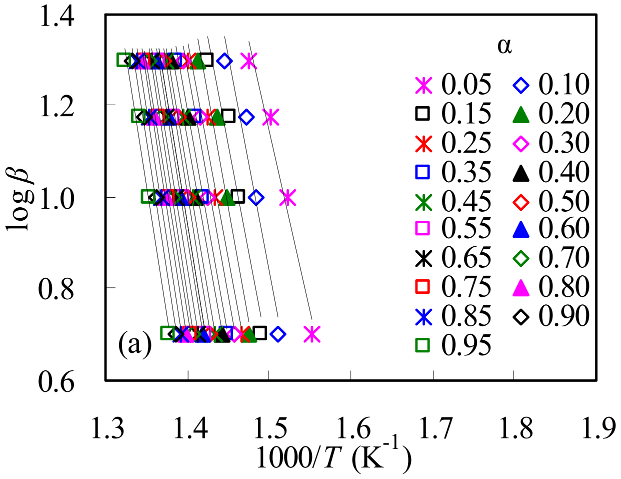

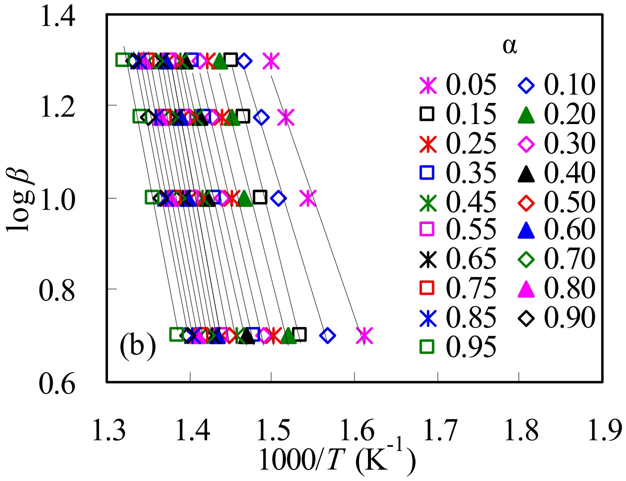

Figure S4. Linear FWO plots of logβ vs. 1000/T obtained for tennis nylon waste in inert N_2 (**a**) and air (**b**).
